# ‘*It’s about time*’: policymakers’ and health practitioners’ perspectives on implementing fertility care in the Gambian health system

**DOI:** 10.1186/s12913-024-10701-0

**Published:** 2024-03-05

**Authors:** Anna Afferri, Susan Dierickx, Haddijatou Allen, Mustapha Bittaye, Musa Marena, Allan Pacey, Julie Balen

**Affiliations:** 1 School of Health and Related Research– ScHARR, The University of Sheffield, Sheffield, UK; 2 Department of Clinical Sciences, Institute of Tropical Medicine, Antwerp, Belgium; 3 Research Centre Gender, Diversity and Intersectionality - RHEA, Vrije Universiteit, Brussel, Belgium; 4 Medical Research Council– MRC Unit The Gambia at LSHTM, Fajara, The Gambia; 5 The Gambia Ministry of Health, Banjul, The Gambia; 6 School of Medicine and Allied Health Sciences, University of The Gambia, Banjul, The Gambia; 7 Faculty of Biology, Medicine and Health, University of Manchester, Manchester, UK; 8 School of Allied and Public Health Professions, Canterbury Christ Church University, Canterbury, UK

**Keywords:** Assisted reproductive technologies, Fertility care, Health policy and practice, Infertility, Private sector, Qualitative research, Reproductive health, The Gambia

## Abstract

**Background:**

Infertility is a major health issue worldwide, yet very few examples of interventions addressing infertility in the Global South have been documented to date. In The Gambia, West Africa, infertility is recognised as a burden and the health authorities have included it in several health policies and the new National Reproductive Health Strategy however, a detailed operationalisation plan for fertility care has not yet been established. Here, we aim to understand and document the factors that influence the implementation of fertility care in The Gambia.

**Methods:**

We conducted 46 semi-structured interviews with policymakers, implementers, and health practitioners in both the public and private sectors from July to November 2021. The interviews were transcribed, anonymised and analysed with NVivo Pro version 1.6.1. The analysis was initially inductive, with themes arising from the coding categorised according to the WHO health systems building blocks framework.

**Results:**

This study identified several barriers to a successful implementation of fertility care in The Gambia, including (i) a lack of routinely collected infertility data; (ii) an absence of financial protection mechanisms for patients, and/or a specific budget for infertility; (iii) limited cooperation between the public and private sectors in the provision of fertility care; and (iv) gaps in fertility care training among health practitioners. Conversely, enablers included: (i) strong national infertility leadership; and (ii) the integration of infertility care within public reproductive health services.

**Conclusion:**

The Gambian health system is not yet in the position to support a comprehensive fertility care package in its public health facilities. Several aspects of the implementation of fertility care must be considered in operationalising the health strategy including the systematic collection of infertility data, fertility awareness, and the provision of specialised fertility care training. Furthermore, a stronger partnership between the public and private sectors must be developed. Given the increasing availability of assisted reproductive technologies in the sub-Saharan Africa region, and the tendency to locate these technologies in the private sector, further research is needed to understand and identify the processes underlying the implementation of fertility care and to foster better integration with the existing health system.

**Supplementary Information:**

The online version contains supplementary material available at 10.1186/s12913-024-10701-0.

## Background

The 1994 International Conference on Population and Development (ICPD) recognised infertility prevention and management as a core component of Sexual and Reproductive Health and Rights (SRHR) [1]. Over the years, this recognition was reiterated by the international community at the World Summit (2005) and as part of the World Health Organisation (WHO) Global Health Strategy (2011). However, despite these international promises, very few concrete examples of interventions addressing infertility in the Global South have been documented to date [2–4].

It can be argued, therefore, that fertility care represents an ‘orphan child’ of the SRHR that has been deprioritised since the ICPD, particularly in resource-limited settings, such as those across sub-Saharan Africa (SSA). Importantly, the drivers behind this apparent de-prioritisation remain highly contested [5]. These include, among others, a predominant discourse on overpopulation [6], limited formal recognition of the impact of infertility on livelihood and wellbeing [7], and a lack of visibility in global health policy arenas [3]. There are some recent signs, however, that infertility awareness among global health stakeholders is improving, with an increased focus on infertility research, policy and practice in some settings [8–10].

Yet, including fertility care in national health agendas is challenging, since in many countries public policies regarding SRHR remain centred on more ‘established’ interventions such as those relating to maternal health, contraceptives, and HIV/AIDS [11–13]. The high cost of assisted reproductive technologies (ARTs) makes it prohibitive in the context of many national health budgets, although other components of fertility care are less costly [14]. Even when infertility is included in health agendas, the implementation of fertility care may, at least initially, result in inequitable access across rural-urban, socioeconomic, education, and gender-based divisions [15]. Turning public policy intentions into a concrete package of actions requires increased engagement with the entire health system (across the public and private sectors) and an improved understanding of the power dynamics, views and positions of policymakers and health practitioners regarding infertility [16, 17].

**Fig. 1:**
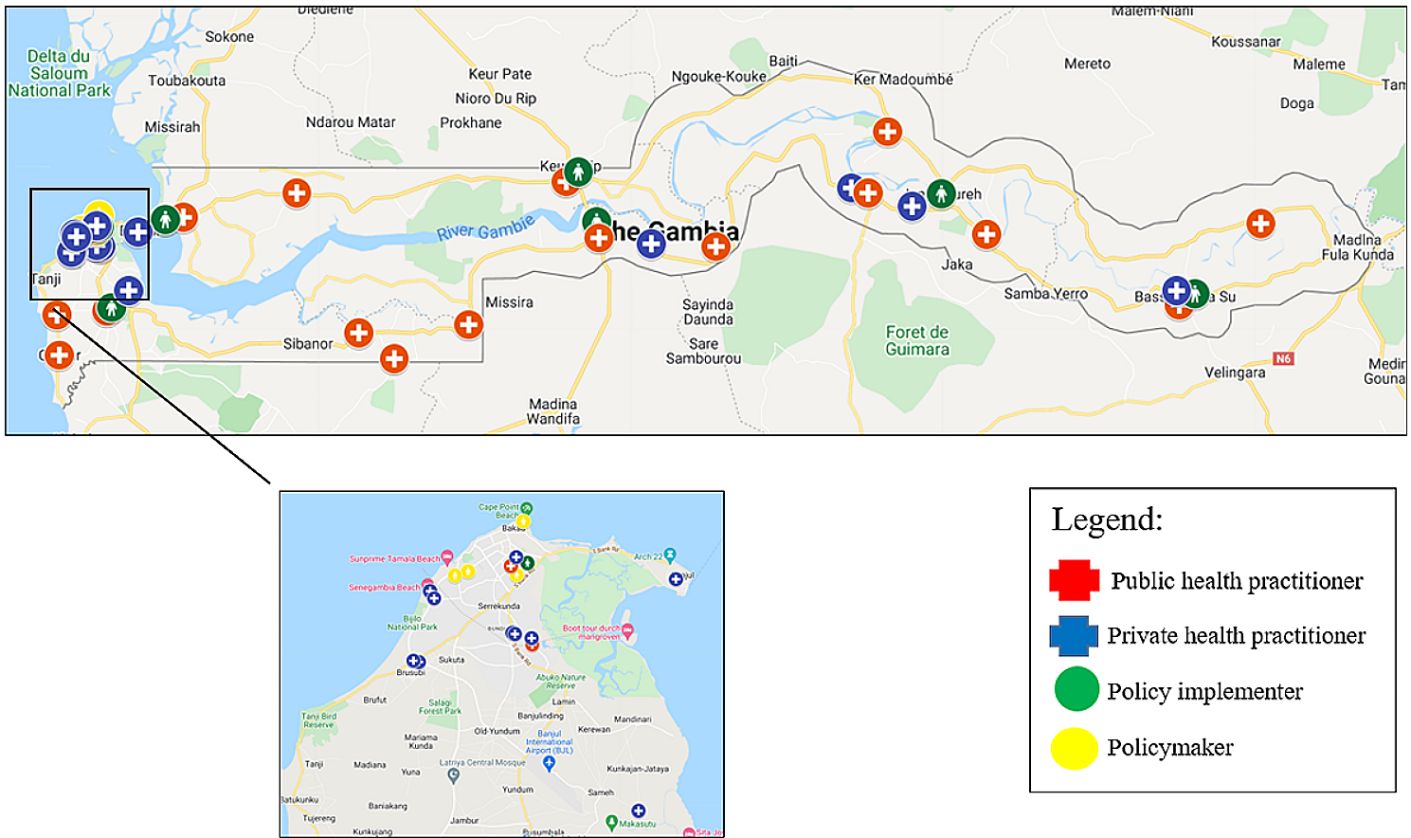
Map of The Gambia, indicating study locations throughout the country (the Greater Banjul area is magnified)^1^

In The Gambia, West Africa (Fig. 1), the Ministry of Health (MoH) recognises infertility as a burden and has taken steps to include it in multiple public health policies and in the new National Reproductive Health Strategy (launched in 2022 though available in draft form at the time of data collection) [18]. However, this recognition will be difficult to maintain without adequate plans to operationalise fertility care. At the end of 2022, the MoH renewed its reproductive health strategic plan and introduced, for the very first time, specific activities to address infertility. These include ‘*provide quality services for the prevention, investigation and treatment of infertility issues…*’, among others [19]. In the country, the most updated data indicate a 12% of infertility prevalence among women seeking pregnancy [20, 21]. Yet, population-based surveys have never been carried out to assess the real impact of infertility, and the reported prevalence is likely to be an underestimation [22]. Many factors can explain the interest of local health authorities in fertility matters, including a strong partnership between the government and academic partners, [4, 23, 24], and ongoing local reproductive activism [25].

Despite (i) the importance of implementing fertility care within health systems, and (ii) the increased global attention given to in scaling-up sustainable health interventions, very few studies in the Global South have been conducted on infertility from a health systems perspective [26, 27]. Here, we aim to understand the factors influencing the operationalisation of fertility care in The Gambia by drawing on the WHO’s health system building block framework [28], and documenting how current national health policies support the implementation of fertility care.

## Methods

### Study design and setting

A qualitative study was conducted between July and November 2021. This study constitutes part of a mixed methods study on fertility care policy and practices in The Gambia [29], and builds on earlier ethnographic research [24, 30–32] The study was carried out in all seven administrative regions of The Gambia, namely the Upper River, Central River, Lower River, North Bank (West and East), and West Coast (1 and 2).

### Sampling and recruitment of participants

Participants were purposely recruited, because of their direct experience with health policy making and implementation and were thus well placed to offer specific insights into the operationalisation of fertility care. The snowballing technique was also applied after each interview. National-level policymakers and policy implementers were selected based on a stakeholder map analysis; healthcare practitioners were recruited from health facilities that were part of a related quantitative cross-sectional study [29]. A total of 52 key informants were contacted, including the MoH at the central and regional levels, representatives of international cooperation agencies, civil society organisations, and healthcare providers. These informants were organised into three categories: (i) policy makers (including the MoH at the central level and international cooperation agencies); (ii) policy implementers (including the regional level of the MoH and civil societies); and (iii) health practitioners, largely in secondary or tertiary care facilities in both the public and private health sectors. Three out of the 52 participants (two private health practitioners and one policy implementer) were unavailable. The remaining 49 participants were recruited over the phone and in-person by a Gambian field assistant. Interviews with health practitioners were conducted in both rural and urban facilities pertaining to both the public and private sectors. The key characteristics of the study participants are shown in Table 1.

**Table 1:** Key characteristics of the study participants

Region	Participant’s profession	Number of participants	Gender
West Coast	Policymaker	*5*	5 male
	Policy implementer	*2*	2 female
	Public health practitioner	*9*	4 male; 5 female
	Private health practitioner	*11*	8 male; 3 female
*Sub-total*		27*	
Upper River	Policy implementer	1	1 male
	Public health practitioner	2	2 male
	Private health practitioner	1	1 male
*Sub-total*		***4***	
Lower River	Policy implementer	1	1 male
	Public health practitioner	2	2 male
	Private health practitioner	1	1 male
*Sub-total*		***4***	
Central River	Policy implementer	3	3 male
	Public health practitioner	3	3 male
	Private health practitioner	2	2 male
*Sub-total*		***8***	
North Bank	Policy implementer	2	2 male
	Public health practitioner	3	3 male
	Private health practitioner	1	1 female
Sub-total		***6***	
*GRAND TOTAL*		***49***	***38 male; 11 female***

* West Coast region is where a large number of the country public and private health facilities are based

### Key informant interviews

Key informant interviews were conducted in English, The Gambia’s official language, with the support of a local field assistant who took notes. Semi-structured interview guides (Additional file 1– Interview guides) were designed to cover themes identified systematically during a qualitative evidence synthesis conducted in 2020 [3]. Interviews were first piloted and then administered through Google Meet (*n* = 3) or in-person (*n* = 43) at the location of each interviewee’s choice to avoid any interference with their regular duties, mostly health facilities and workplace. All participants received an information sheet and interviews were audio and/or video recorded with interviewees’ informed consent and permission and lasted between 15 and 60 min (with an average of 30 min). The Consolidated Criteria for Reporting Qualitative Studies (COREQ) checklist was used to document the study quality (Additional file 2– **COREQ checklist**) [33].

### Data analysis

Interviews with key informants were transcribed *verbatim* prior to inductive analysis and according to the principles of thematic coding [34]. Coding was performed by the lead author using QSR International’s NVivo Pro qualitative software, version 1.6.1 (released in 2020). The sentences extracted from the transcripts were coded with nodes (Additional file 3– Thematic analysis codebook), and later categorised into the health system building blocks framework, namely: (i) leadership and governance; (ii) health information system; (iii) health financing; (iv) service delivery; (v) medicines and technologies; and (vi) health workforce [28, 35, 36]. The selection of this framework was driven by the purpose of identifying factors influencing the implementation of fertility care in the Gambian health system “*to benefit public health through [a] more effective, efficient, equitable and acceptable system*” [37] and testing the readiness of the Gambian public health system to scale-up fertility care in the country [18].

### Ethical approval

Ethical approval was obtained from The Gambia Government and Medical Research Council (MRCG) at the London School of Hygiene and Tropical Medicine Joint Ethics Committee (Reference 22,446) and the University of Sheffield– School of Health and Related Research (ScHARR) Research Ethics Committee (Reference 03785–038109). Written informed consent was obtained from all respondents prior to the beginning of the data collection.

## Results

### Leadership and governance

All respondents agreed that the MoH would pay attention to fertility care and the composition of the leadership team was identified as a key driving force behind this decision’*“…It’s about time [to include fertility care] and it’s about having the people, the right people with the right mind at the right place…’ (Male, public health practitioner - West Coast).* However, despite of this recognition of the timeliness and importance of including fertility care in the National Health Policy, most respondents also identified potential threats to the implementation of the policy, particularly with respect to the fertility care component:

*‘It is not going to be an easy thing. It is going to cost a lot of energy to invest. Because it’s quite a ladder, what you’re naming here, from education to healthcare system, it is a lot’ (Female, private health practitioner– West Coast)*.

Coordination among stakeholders involved in fertility care (public, private and international partners) was desired by respondents but was criticised as missing at various levels. Private healthcare workers, in particular, felt that the MoH has not involved them in policy and/or decision-making processes and they expressed a wish for a closer collaboration:

*‘The Ministry of Health gives no help to this private clinic, therefore this private clinic gives no help to the Minister of Health. They have never visited this center. They have never called us for any policy decision. They have never consulted us in anyway. This is not politics but just courtesy’ (Male, private health practitioner– West Coast)*.

*‘The coordination is done in all other areas with the Ministry of Health but [for infertility] they are not properly coordinated. The coordination should start from the Ministry as, it has an ownership role’ (Male, policymaker– West Coast)*.

Similarly, the involvement of international partners was recognised as a key gap but also an area for improvement *‘Many partners are here, if they are faced by the Ministry [of Health], I believe they will come in’ (Male, policy implementer– Lower River).*

Fertility awareness activities, factors influencing the perceived importance of infertility, were rarely reported. To this end, respondents were unable to recall any major initiatives related to fertility except for a few events such as occasional television, radio shows and public marches aiming to increase awareness of infertility. This observation was even more pronounced in rural areas:

*‘But infertility is something that I have not seen been discussed openly. I have not seen any team come around to do any activities, programs, it’s something that I have not seen around’ (Male, public health practitioner–– West Coast)*.

### Health information system

Data on infertility are not routinely captured or officially requested by the MoH. The majority of respondents reported that the current health management information system (HMIS) lacks dedicated space to collect information on infertility [29]. Health practitioners noted that this resulted in them having to enter data from patients with infertility under unspecified categories which they found problematic:

*‘…infertility might not be reported or it is reported as “Others”. Some of the conditions that are not reflecting in the DHMIS [district health management information system] are all gathered and reported as ‘Others’. So in these others you may not be able to differentiate what is what” (Male, public health practitioner– North Bank)*.

*‘In the district health management information system [DHMIS] where the data is collected, there is no area talking about infertility’ (Female, policy implementer– West Coast)*.

*‘Usually what we are requesting [from the health facilities], we get. We have an electronic system where we can get the data we want, but for infertility there is none, we are not requesting that, so we are not getting it’ (Male, policymaker– West Coast)*.

### Health financing

The absence of any funding earmarked for fertility care was a serious concern for both policymakers and healthcare professionals. The general view was that the MoH should have a dedicated and detailed budgetary allocation to implement fertility care, and that should also interact more with in-country international development partners and the private sector, to co-fund selected fertility care interventions:

*‘…the only thing we can say is that the Ministry of Health needs to put [fertility care] in the budget. Whether there will be money for it or not, that question is very difficult for us to answer’ (Males, policy implementer - Central River)*.

*‘… [fertility care] would have budget implications, maybe to an extent the government might try to see how best to negotiate any help from partners’ (Male, policy implementer, Upper River)*.

A few participants proposed the introduction of fertility care under the current health insurance scheme to help decrease out-of-pocket expenditures for fertility care:

*‘…if they can introduce in the policy a health insurance scheme, so who cannot afford to buy these drugs can use their health scheme card to pay in any pharmacy, I think that will help a lot’ (Female, public health practitioner, West Coast)*.

### Service delivery

The public health facilities that provide infertility care, have largely been integrated with other reproductive health services and delivered within family planning or gynecology clinics; a slightly different picture has emerged from the private sector, where standalone infertility services are available in specialised clinics and on designated days (for further details see [3]. Barriers in health seeking and low engagement with diagnostic services, especially among men compared to women, were reported as major issues for successful implementation of fertility care interventions. A lack of men’s involvement in infertility services was cited as one of the central challenges to the successful delivery of infertility services. The respondents suggested that better care for men with fertility concerns will require changes in attitudes and perceptions of male reproductive health in society:

*‘…the belief is that as long as the man is having a normal erection, has a normal intercourse, and can ejaculated, everything’s fine. So since everything is fine, they think there is no problem. Sometimes it’s challenging to make them understand’ (Male, public health practitioner - West Coast)*.

*‘We also want the men to help contribute in the management of infertility, if you send your woman or your wife in the clinic and you are not there, that also is difficult, it hinders the treatment. So, you also must appeal to the men’ (Male, public health practitioner– Central River)*.

Respondents noted that there is currently no fertility assessment tool available for use in public or private facilities. When asked specifically about FertiStat [38–40], a self-assessment fertility tool to rapidly evaluate fertility status, and counsel men and women toward the most appropriate course of action, none of the participants had any knowledge of it:

*‘…I never had the opportunity to get access to it [FertiStat]…I have never used it before. I am not sure if it is a general guiding protocol from the Ministry of Health, but I never come across something like that…’ (Male, policy implementer– North Bank)*.

Respondents claimed that the private sector in The Gambia provides a broader range of infertility diagnostic investigations and treatment options, than do those in public facilities ‘*Infertility care is provided more in the private clinics than in the public sector’ (Male, public health practitioner, West Coast).* However, they also noted that the high costs of accessing private care, which are born by patients through out-of-pocket expenditures, pose an equity issue in terms of lack of affordability among many of those in need: *‘Infertility for the Gambians is costly: they have to pay for consultation, they have to pay for scan, and they have to pay for medication’ (Male, private health practitioner– West Coast).*

A partnership model between the public and private sectors for the delivery of fertility care is an option that was said to not yet be fully developed or utilised in The Gambia *‘…the coordination with the private sector is not very strong like in other countries, that’s not happening in The Gambia…’ (Male, policymaker– West Coast).*

### Medicines and technologies

Participants shared concerns about the implementation of fertility care in the public sector because of health system challenges such as shortage of medications and unavailability of certain equipment required for infertility investigations and treatment. Furthermore, the aforementioned ability of the private sector to respond better to fertility needs appears to have altered the referral system. In this regard, public health practitioners cited that despite having a preference for referring patients primarily within the public health system, the unavailability of fertility specialists and long waiting times, compel them to direct patients toward private care:

*‘…the drugs are not available, because the Ministry of Health is looking at what is called essential medicines and in these, infertility treatment is not captured. So, it means patients have to go and buy them… (Male, policy implementer– Lower River)*.

*‘…I refer to Banjul [teaching hospital] but for infertility they refer here. Why? Because even the IUI is not there, the drugs for [ovarian] stimulation are not the better [ones], I have all of them’ (Male, private health practitioner– West Coast)*.

Participants also recognised how expensive infertility investigations and treatment are, and how this may pose a problem for both access and discontinuation of care:

*‘…And financially, most of people don’t have money. Poor people in the sub-region can’t afford to do some of these tests. So the moment you add them, you won’t see back again, we don’t see them again…they just go…’ (Male, public health practitioner– West Coast)*.

### Health workforce

Only a few of the participants reported being trained in infertility management more generally, specifically in ART. A majority of participants noted that little information on infertility was obtained during their formative years (nursing school or university) and/or during their clinical practice (i.e. on-the job training). Most of the health practitioners interviewed described that to safely and fully implement fertility care, they would require appropriate training. This point was also highlighted as one of the challenges to implementing fertility care within the context of the new National Health Policy:

*‘…I used to attend the infertility clinic in Banjul. Some of the [infertility] knowledge is captured from your colleagues, but other coming from your own reading and what you have learned from the medical school…’ (Male, public health practitioner– North Bank)*.

*‘…Healthcare staff should be trained to identify infertility and also to be able to treat infertility even at public facility-level…’ (Male, policy implementer -Lower River)*.

Furthermore, a limited number of health practitioners were trained in ART abroad, yet frustration levels were reportedly high among this cohort as they were unable to capitalise on their training, since ARTs are not currently available in The Gambia:

*‘…It’s really frustrating, because after the training in India, you come back with all that knowledge and all that skills to apply to help people here and you find nothing. And then the government is not encouraging. They don’t provide the background as far as to do those things. We came back…but I’m applying the basic, basic skills…’ (Male, public health practitioner– West Coast)*.

Finally, respondents revealed that the deployment and retention of human resources for health is a major concern for the implementation of fertility care. This divide was even larger between health providers posted in rural facilities:

*‘…personnel and skills will be a challenge [to implement fertility care], especially in the rural Gambia because we are under staffed…we need a gynaecologist to take care of certain kind of things or a medical officer…’ (Male, public health practitioner, Central River)*.

*‘…to address infertility, the Ministry of Health should also identify consultants [doctors] to work in the interior of the country because 95% of the people affected by infertility are from the rural communities.…’ (Male, private health practitioner– Upper River)*.

### People-centred system

People are often listed as being at the core of a health system. The term ‘people’ is intended to represent individuals, families, communities, civil society, consumers, patients, and healthcare providers that through knowledge, attitudes, behaviours, and practices influence the demand and supply of services and the health system itself.

Despite an analysis of the demand for infertility services being out of the scope of this study, respondents frequently noted that infertility is seen as a highly stigmatised ‘female’ problem that is deeply rooted in sociocultural beliefs, especially gender norms and expectations, and that this ‘bias’ greatly impacts the demand for infertility services. Childless women in The Gambia were said to face marital and familial discord as relatives frequently initiate gossip in the household and instigate the husband’s decision to remarry to procreate:

*‘…Everybody will blame the woman for not having a child. That stigma is always there with the woman. When a woman don’t have a child in the family, in the compound, you hear so many things. So many bad things from the family members, from your co-wives, from the in-laws, from everywhere…’ (Female, private health practitioner– West Coast)*.

*‘…So an extent, some family members can try to get problem saying ‘This one is not good to give us anything’ suggesting the husband should take a second wife. They fail to understand that certain times the fault is not on the woman but on the man…’ (Male, policy implementer– Upper River)*.

Informal medicine is practiced in The Gambia with traditional healers and *marabouts* often being the first points of care contact. Some childless couples undertake traditional treatment for several years before reporting their fertility concerns at a health facility, and this delay in seeking formal care usually hampers their financial management as well as their reproductive health outcomes:

*‘…the first port of call would be the traditional healer…then the spiritual healer, sometimes they are asked to pay a lot in kind or in cash…Unfortunately, the flourishing of the traditional treatment in the country has affected a lot of reproductive health issues, notwithstanding infertility as well…’ (Male, policymaker– West Coast)*.

*‘…first they go to traditional healers. Until they’ve exhausted all those places that is the time they normally come back [seeking help] in the facility…’ (Male, public health practitioner, North Bank)*.

## Discussion

This study illustrates the importance of having a clear implementation plan to support fertility care intentions and to help embed fertility care in the Gambian public health system. Some of the implementation challenges expressed by the participants reflect wider barriers faced by the Gambian health system [41, 42], while others are specific to fertility care. These factors should be carefully taken into account when planning and operationalising fertility care. While challenges were noted across each of the health system ‘building blocks’, some appeared to be more critical than others, including: (i) health information system; (ii) health financing; (iii) health governance; and (iv) health workforce.

### The health information system and collection of infertility data

Given that the current data are not captured or transmitted within the Gambian health system, and that in the few instances of collection, they are aggregated with other health conditions, the likely implications are that a real picture of the demand for and access to infertility services is missing. Having reliable and accurate data on infertility is an important step for the health system both for statistical purposes and for attracting attention and international interest as well as funding and further research in the area [4, 43, 44]. A priority for Gambian policymakers should therefore be the revision of the health information system form with a space allocated to the collection of infertility data [45].

### Financial protection for people with infertility and a service-specific budget

Health insurance coverage, providing a pathway toward universal health coverage (UHC) [44], is very low in The Gambia. Recent research has estimated that only 4% of Gambians are protected by an insurance scheme (41) and currently, the government subsidises only civil servants. In November 2021, a national health insurance bill was passed with the intention of periodically reviewing the list of health conditions covered by the insurance scheme (29). It is unlikely that fertility care will be covered through the health scheme in the near future, but as suggested by study participants, this could be considered an option in the longer-term to help reduce out-of-pocket expenditures.

Allocation of a budget for medications and equipment is central to successful fertility care implementation and service delivery. In this regard, drugs such as clomiphene citrate, an inexpensive drug used to stimulate ovarian response [46] are not currently available in the public sector despite being listed as MoH essential medicines.

Ensuring availability of medications in the public sector may further decrease out-of-pocket expenditures for fertility care and could thereby also contribute to improving fertility-equity in The Gambia.

### Improving public-private partnerships and collaboration in fertility care

The presence of private clinics providing fertility care has reshaped the referral pathway of the Gambian health system. As a result, some patients who can afford treatment prefer to be referred to private clinics or to directly visit private health clinics by themselves. As in other countries [47, 48], private care for infertility is an emerging market in The Gambia. This embodies the limited availability of infertility services in public facilities and the high demand for services that do not stop those in need from accessing costly treatments [14, 49]. As an example of an emerging market, during the data collection phase, the study team learnt of two new private fertility clinics. Both are intending to provide ART as soon as they can employ an embryologist, and both are now part of the referral system in the coastal area of the Western region. This proliferation of private clinics aiming to provide fertility care, as in other parts of SSA, highlights the urgent need for consideration of international or national guideline adoption, to avoid the risks associated with unregulated provision.

Another issue emerging within the Gambian health system, is the absence of a comprehensive census of private clinics operating in the country. Mapping the private sector in all its aspects (service provision, distribution and geographic coverage) is required to maximise the resources currently available [41]. Moreover, the interaction and collaboration between the public and private sectors are presently very limited and, in certain instances, even conflictual. Care needs to be taken with regard to the intertwining of public health priorities and policies, and the objectives and drivers of the market-oriented private sector [50]. Perhaps it is premature for the Gambian public health system to budget for the first public Assisted Conception Unit, but discussions should be initiated with the private sector where readiness for ART is much more advanced. Currently, different low-cost medically assisted reproduction (MAR) procedures are available which are potential solutions for increasing access to infertility treatments. These include, among others, intravaginal culture [51], mild stimulation protocols [52], and simplified egg culture [53, 54]. However, careful consideration must be taken into account because studies on the long-term safety of these affordable procedures remain limited [55].

### Development of an infertility-responsive health workforce

The Gambia is faced with a chronic shortage of human resources for health (HRH) [41]. The development of HRH is not the only issue that the public health system is facing: both the production and retention of the health workforce, above all in rural areas, further affect the delivery of health services, including the provision of infertility services. While more than 40% of the Gambian health facilities offering infertility services are located in rural areas [29], living and school conditions in those settings are particularly difficult for health providers who prefer to work in urban areas where the management of work and family life is easier [56].

Currently, specialised training in infertility management and embryology is provided through a scholarships requiring selected Gambian doctors to travel abroad [57] and this training has to date supported approximately 6 medical doctors (including co-author MM). Further strengthening the Gambian Higher Education sector and seeking technical and long-term capacity strengthening partnerships with countries in the region (i.e. Nigeria, Senegal and Ghana) could expand the pool of health providers trained in fertility care.

### Study limitations

This qualitative study aimed to provide an in-depth understanding of the factors influencing the implementation of fertility care within the Gambian health system. Previous anthropological research in the rural and urban areas of the West Coast region, provided a comprehensive overview of the health-seeking behaviour of people with fertility challenges; for this reason, we have omitted the perspectives of patients with infertility and /or community members from this study [24, 31, 32]. However, we strongly acknowledge the importance of community voices including male voices and those of fertility care patients. We also noted that while embarking on research focused on infertility in a low-income country, it is imperative to acknowledge the inherent complexities and sensitivities surrounding this subject matter. The authors of this study identified a diverse set of values, cultural perspectives, and educational backgrounds that influenced the framing of the study and interpretation of the data. They came from multiple countries (western and African), and from multiple disciplines (anthropology, health system research, andrology, medicine), and had lived experiences of infertility or of fertility care provision in The Gambia. Lastly, the views of the key participants in this study may not be representative of all fertility experts or broader health stakeholders because some have not participated in the study or were unavailable at the time of data collection. Overall, however, the study was conducted across all regions of The Gambia, in public and private facilities at the secondary and tertiary levels, representing a comprehensive set of facilities throughout the country.

## Conclusion

The Gambian public health system is not yet in a position to support a comprehensive fertility care package in its health facilities, but by including fertility care within its renewed health policy, it has laid the foundation for potentially improving infertility management in the future. This study identified several aspects of the implementation of fertility care that must be considered before the operationalisation of the strategy. First, a fertility care policy, implementation plan, and budget must be acknowledged within the different levels of the health system, thereby avoiding the tendency to develop a top-down approach without any discussion with policy implementers or health providers, who are ultimately responsible for putting in practice policy interventions; second, infertility data should be collected, transmitted, and shared throughout the health system in a systematic manner to permit evidence-informed policy making; third, the skills of health providers need to be updated in terms of specialised fertility care training and according to their level of care; fourth, a more robust partnership with the private sector must be built because, currently private clinics are the main providers of infertility services in the country; finally, fertility care needs a dedicated strategic plan in which vision, outputs, outcomes and funds for infertility are carefully considered and allocated. Given the increasing availability of ARTs in several countries in the SSA region and the tendency to locate these ARTs in the private sector, further research is needed to understand and identify the processes underlying the implementation of fertility care and to foster better integration with the existing health system.

### Electronic supplementary material

Below is the link to the electronic supplementary material.

Supplementary Material 1: A1? Interview guides

Supplementary Material 2: A2? COREQ checklist

Supplementary Material 3: A3? Thematic analysis codebook

## Data Availability

The datasets generated and analysed during the current study are not publicly available due to the sensitive nature of the study, but they can be made available upon reasonable request made to the lead author.

## Footnotes

1 Figure 1 created by the lead author using Google Maps

## Abbreviations

ART: Assisted Reproductive Technologies
HRH: Human Resources for Health
ICPD: International Conference on Population Development
MOH: Ministry of Health
SRHR: Sexual and Reproductive Health and Rights
UHC: Universal Health Coverage
WHO: World Health Organisation
