# Supplementary material for: ‘It’s about time’: policymakers’ and health practitioners’ perspectives on implementing fertility care in the Gambian health system
Source: BMC Health Serv Res. 2024 Mar 5;24:282. doi: 10.1186/s12913-024-10701-0 (PMC10916196; doi:10.1186/s12913-024-10701-0)
Supplement: Supplementary file 1 — Supplementary Material 1: A1? Interview guides [file 12913_2024_10701_MOESM1_ESM.pdf]

Interview guide for Policymakers

Could you please tell me about your position?

a. For how long have you been in this post?
b. In relation to sexual and reproductive health activities, what kind of support do you provide?
c. What are the top five priorities of sexual and reproductive health in The Gambia?
d. Do you think infertility is a health priority in The Gambia? Why (why not)?
e. In the past and also now, there was an involvement from high cadres of the government in infertility issues. How do you think this has influenced fertility care and infertility services provision in the country?

1. Leadership & Governance

a. In your opinion, how does the national reproductive health policy fulfil the needs of the infertile women? How for infertile men?
b. Do you think it is necessary to include fertility care in the national reproductive health policy? Why (why not)?
If yes, what mechanism could facilitate the inclusion?

2. Service delivery/Partnership

a. What is in place to facilitate patients' to access infertility services? (*Probe for regulations, policy, skilled providers, health insurance scheme, etc.*)
b. Is there any difference between the infertility care provided by the public and private sector?
    a. If Yes, which one?
    b. How does this influence the provision of infertility care?
c. How does the government interact with organizations/foundations/institutions/private sectors involved in infertility services? (*Probe for: coordination meetings, infertility awareness activities, annual workshops, etc.*)
d. How WHO Gambia or other UNs agencies, involved in reproductive health, support the government concerning fertility care?
e. What partnership, if any, concerning infertility services is in place between the public and private sector?
f. What mechanisms are in place to ensure safe standard procedures and quality control for infertility treatments? (*probe also for private sector*)
g. Is the Gambia member of any infertility care alliance/association (*Probe for*: *African Infertility Alliance, International Infertility Alliance*)?

3. Essential medicines

a. How affordable are Assisted Reproduction Technologies (ART), in The Gambia? Please explain.
b. What mechanisms are or should be put in place to allow infertile couples to benefit from ART?
c. Which mechanism is established to ensure the provision of drugs, equipment and commodities for the provision of infertility services? Could you explain?

4. **Workforce**

a. In The Gambia, are there any health professionals who can provide infertility services?
   If yes, what kinds of services does this include?
b. Who are the health professionals involved in the provision of infertility services?
c. How are these providers trained to manage infertility issues?

5. **Financing**

a. What are the main sources of funding for fertility care? (*Probe for*)
   Govt
   International donors
   Private sector
   Faith-based organisations/Foundations
   Out-of-pocket (family-patient contribution)

b. Within the health budget, what is the proportion allocated for the provision of infertility care?
   Do you think this proportion might change in the future? Why (why not)?

6. **Health Information System**

   a. What mechanisms are in place to report on infertility cases?
   b. Are those mechanisms different from the routine data collection?
   c. How frequently is infertility data reported? (*Probe for monthly, quarterly, annual*)
   d. What challenges the actual monitoring system has in reporting data on infertility?

7. **Overall perspective in infertility care integration**

What do you believe are some of the most important factors that might facilitate the inclusion of fertility care into the Gambian reproductive health policy?

   a. What are the major challenges and/or constraints to include fertility care into the reproductive health policy in the Gambia?
   b. Do you think it is necessary to include fertility care in the national reproductive health policy? Why (why not)?

Do you have any other information, concerning fertility care and infertility services, you would like to share with me?

Interview guide for Policy Implementers

<u>*Patients' organisations/associations*</u>

Could you tell me about your position?

a. For how long have you been in this post?
b. In relation to sexual and reproductive health, what kind of support your organisation provides? (*Probe for infertility awareness, infertility counselling, media information, community sensitisations, etc*.)

I would like to ask you some questions about infertility in The Gambia.

1. In your opinion, do you think infertility is a health priority in The Gambia? Why (why not)?
2. In the past and also now, there was an involvement from high cadres of the government in infertility issues. How do you think this has influenced fertility care and infertility services provision in the country?
3. How do people with infertility access infertility services?
   a. Which barriers the patients encounter to access infertility services? (*Probe for gender, costs, etc.)*
   b. What is in place to facilitate patients' to access infertility care? (*Probe for regulations, policy, skilled providers, health insurance scheme, etc.)*
4. Is there any difference between the infertility care provided by the public and private sector?
   a. If Yes, which one?
   b. How does this influence the provision of infertility services?
5. How affordable are Assisted Reproduction Technologies (ART), in The Gambia? Please explain.
   a. What mechanisms are or should be put in place to allow infertile couples to benefit from ART?
6. Do you receive any support from national or international organisations when it comes to fertility care? Explain a little bit more about the kind of support received, if any.
7. How would you describe the interaction between the Government and your organization concerning fertility?
8. What do you believe are some of the most important factors that might facilitate the inclusion of fertility care into the Gambian reproductive health policy?
   a. What are the major challenges and/or constraints to include fertility care into the reproductive health policy in the Gambia?
   b. Do you think it is necessary to include fertility care in the national reproductive health policy? Why (why not)?
9. How do you see the future of your organization in the provision of fertility support to the Gambian citizens?

Do you have any other information, concerning infertility, you would like to share with me?

National and international for profit and non-for-profit organisations

Could you tell me about your position?

a. For how long have you been in this post?
b. In relation to sexual and reproductive health, what kind of support your organisation provides? (*Probe for infertility care including ART, infertility awareness, media information, community sensitisations, infertility counselling, etc*.)
c. What are the top five priorities of sexual and reproductive health in The Gambia?

I would like to ask you some questions about infertility in The Gambia.

1. In your opinion, do you think infertility is a health priority in The Gambia? Why (why not)?
2. In the past and also now, there was an involvement from high cadres of the government in infertility issues. How do you think this has influenced fertility care and infertility services provision in the country?
3. To the best of your knowledge, how infertile couples get access to infertility services?
   a. Which difference exists between women and men getting access to infertility services in The Gambia?
   b. What is in place to facilitate patients' to access infertility services? (*Probe for regulations, policy, skilled providers, health insurance scheme, etc.*)
4. Is there any difference between the infertility care provided by the public and private sector?
   c. If Yes, which one?
   d. How does this influence the provision of infertility care?
5. How affordable are Assisted Reproduction Technologies (ART), in The Gambia? Please explain.
   b. What mechanisms are or should be put in place to allow infertile couples to benefit from ART?
6. (*When relevant*) In your opinion, does the current national reproductive health policy fulfil the needs of the Gambian infertile citizens?
   a. Do you think it is necessary to include fertility care in the national reproductive health policy? Why (why not)?
   b. If yes, what mechanism could facilitate the inclusion?
7. How would you describe the interaction between the Government and your organization concerning infertility?
   a. What partnership, *if any*, concerning infertility services is in place between your organisation and the Government?
   b. What are your tasks in this partnership?
   c. What are the Government tasks?
   d. How is this different for the public or private sector?
8. Is there a technical/coordination working group on infertility? If Yes,
   a. Who is part of it?
   b. What are the responsibilities of this working group?
   c. How often do you meet?

   d. Who is leading the group?
9. Do you receive any national or international support when it comes to fertility care? Explain a little bit more about the kind of support received, if any.
10. What do you believe are some of the most important enabling factors that might facilitate the inclusion of fertility care into the Gambian reproductive health policy?
    a. What are the major challenges and/or constraints to include fertility care into the reproductive health policy in the Gambia?

Do you have any other information, concerning infertility, you would like to share with me?

Interview guide for Health Practitioners

Please tell me about your position in this facility.

a. For how long have you been in this post?
b. In relation to sexual and reproductive health activities, what kind of support do you provide?

1. Do you think the provision of infertility services is a priority for your facility and the catchment population? Tell me more about it.
2. Concerning the delivery of infertility services in your facility, what are your thoughts about infertility be integrated with other reproductive health services?
3. Overall, where would be the best place to position infertility services in the sexual and reproductive health programme? (*Probe for*)
    a. *Maternal and newborn care programmes*
    b. *Fertility clinic*
    c. *Ob/Gyn clinic*
    d. *Andrology clinic*
    e. *Urology clinic*
    f. *Family planning/contraception clinic*
    g. *STIs clinic*
    h. *HIV clinic*
    i. *Community health interventions*
4. What issues are you facing with trying to help patients living with infertility?
5. Which training the health staff (you) received in fertility care?
6. Do you have any partnership/support to carry out fertility care in this facility? Tell me about it. (*Probe for MoH support, private organisations, community groups, etc.*)
    a. If yes, how does the MoH support the provision of infertility services in this facility?
7. How the referral pathways are organised, for infertile patients, between levels of care? (*Probe from primary to secondary to tertiary AND public to private or vice versa)*
8. Where infertile patients are referred to and from? (*Probe for*)
    a. *Pharmacy Providers*
    b. *Complementary medicine providers*
    c. *Traditional healers or herbalists*
    d. *Public specialised medical diagnostic and treatment services*
    e. *Private specialised medical diagnostic and treatment services*
    f. *Mental health and psychological support services*
9. What kind of linkages are available with the public (or private) clinics concerning infertility services?
10. Is this facility registered as part of Africa Network and Registry for Assisted Reproductive Technology - ANARA?
    a. Is this facility registered with any ART monitoring organisation (*Probe for ICMART*)?
11. What difference exists between the public and private sector in the provision of infertility care in your country?

12. In your opinion, what are some of the most important factors that might facilitate the inclusion of fertility care into the Gambian reproductive health policy?
    a. What are the major challenges and/or constraints to include fertility care into the reproductive health policy in the Gambia?
    b. Do you think it is necessary to include fertility care in the national reproductive health policy? Why (why not)?
13. (*Optional*) What would you see done differently in the way infertility services are delivered in your facility?

Do you have other information concerning infertility services, you would like to share with me?
