# Supplementary material for: ‘It’s about time’: policymakers’ and health practitioners’ perspectives on implementing fertility care in the Gambian health system
Source: BMC Health Serv Res. 2024 Mar 5;24:282. doi: 10.1186/s12913-024-10701-0 (PMC10916196; doi:10.1186/s12913-024-10701-0)
Supplement: Supplementary file 2 — Supplementary Material 2: A2? COREQ checklist [file 12913_2024_10701_MOESM2_ESM.docx]

**Consolidated criteria for reporting qualitative studies (COREQ): 32-item checklist**

Developed from: Allison Tong, Peter Sainsbury, and Jonathan Craig. *Consolidated criteria for reporting qualitative research (COREQ): a 32-item checklist for interviews and focus groups*. International Journal for Quality in Health Care. Volume 19, Issue 6, December 2007, Pages 349–357. <https://doi.org/10.1093/intqhc/mzm042>

| **No. Item** | **Guide questions/description** | **Reported on Page #** |
| --- | --- | --- |
| **Domain 1: Research team and reﬂexivity** | | |
| *Personal Characteristics* |  |  |
| 1. Interviewer/facilitator | Which author/s conducted the interview or focus group? | **AA** |
| 2. Credentials | What were the researcher’s credentials? E.g. PhD, MD | **MPH (not reported on page)** |
| 3. Occupation | What was their occupation at the time of the study? | **PhD student (not reported on page)** |
| 4. Gender | Was the researcher male or female? | **Female** |
| 5. Experience and training | What experience or training did the researcher have? | **AA has conducted qualitative research for over 8 years** |
| *Relationship with participants* |  |  |
| 6. Relationship established | Was a relationship established prior to study commencement? | **None** |
| 7. Participant knowledge of the interviewer | What did the participants know about the researcher? e.g. personal goals, reasons for doing the research | **A participant information sheet was distributed to each participant prior the interview (page 8)** |
| 8. Interviewer characteristics | What characteristics were reported about the interviewer/facilitator? e.g. Bias, assumptions, reasons and interests in the research topic | **None** |

| **Domain 2: study design** | | |
| --- | --- | --- |
| *Theoretical framework* |  |  |
| 9. Methodological orientation and Theory | What methodological orientation was stated to underpin the study? e.g. grounded theory, discourse analysis, ethnography, phenomenology, content analysis | **Grounded theory, thematic coding, ethnography**  **(page 6)** |
| *Participant selection* |  |  |
| 10. Sampling | How were participants selected? e.g. purposive, convenience, consecutive, snowball | **Purposive and snowballing (page 6)** |
| 11. Method of approach | How were participants approached? e.g. face-to-face, telephone, mail, email | **Phone, email, and face-to-face (page 7)** |
| 12. Sample size | How many participants were in the study? | **52 (page 7)** |
| 13. Non-participation | How many people refused to participate or dropped out? Reasons? | **3 (page 7)**  **Not available** |
| *Setting* |  |  |
| 14. Setting of data collection | Where was the data collected? e.g. home, clinic, workplace | **Health facilities and workspace (page 8)** |
| 15. Presence of non-participants | Was anyone else present besides the participants and researchers? | **Yes, a note taker (page 8)** |
| 16. Description of sample | What are the important characteristics of the sample? e.g. demographic data, date | **Majority was male and from the West Coast (page 7)** |
| *Data collection* |  |  |
| 17. Interview guide | Were questions, prompts, guides provided by the authors? Was it pilot tested? | **Yes (supplementary information S1)** |
| 18. Repeat interviews | Were repeat interviews carried out? If yes, how many? | **No** |
| 19. Audio/visual recording | Did the research use audio or visual recording to collect the data? | **Yes (page 8)** |
| 20. Field notes | Were ﬁeld notes made during and/or after the interview or focus group? | **Yes (page 8)** |
| 21. Duration | What was the duration of the interviews or focus group? | **Between 15 and 60 minutes (page 8)** |
| 22. Data saturation | Was data saturation discussed? | **No** |
| 23. Transcripts returned | Were transcripts returned to participants for comment and/or correction? | **No** |
| **Domain 3: analysis and ﬁndings** | | |
| *Data analysis* |  |  |
| 24. Number of data coders | How many data coders coded the data? | **1 (page 8)** |
| 25. Description of the coding tree | Did authors provide a description of the coding tree? | **No** |
| 26. Derivation of themes | Were themes identiﬁed in advance or derived from the data? | **Both (page 6)** |
| 27. Software | What software, if applicable, was used to manage the data? | **Yes. QSR International NVivo Pro (page 8)** |
| 28. Participant checking | Did participants provide feedback on the ﬁndings? | **No** |
| *Reporting* |  |  |
| 29. Quotations presented | Were participant quotations presented to illustrate the themes/ﬁndings? Was each quotation identiﬁed? e.g. participant number | **Yes (pages 10-17)** |
| 30. Data and ﬁndings consistent | Was there consistency between the data presented and the ﬁndings? | **Yes** |
| 31. Clarity of major themes | Were major themes clearly presented in the ﬁndings? | **Yes (pages 10-21)** |
| 32. Clarity of minor themes | Is there a description of diverse cases or discussion of minor themes? | **Yes (pages 17-21)** |
