# Supplementary material for: ‘It’s about time’: policymakers’ and health practitioners’ perspectives on implementing fertility care in the Gambian health system
Source: BMC Health Serv Res. 2024 Mar 5;24:282. doi: 10.1186/s12913-024-10701-0 (PMC10916196; doi:10.1186/s12913-024-10701-0)
Supplement: Supplementary file 3 — Supplementary Material 3: A3? Thematic analysis codebook [file 12913_2024_10701_MOESM3_ESM.docx]

**S2 – Thematic analysis codebook**

| ***Codes*** | ***Files*** | ***References*** |
| --- | --- | --- |
| **DIFFERENCES PUBLIC - PRIVATE** | **24** | **90** |
| Availability of drugs | 5 | 5 |
| Availability of equipment | 7 | 11 |
| Availability of infertility services | 11 | 13 |
| Brain drain from public to private | 1 | 1 |
| Confidentiality | 1 | 1 |
| Cost of services | 11 | 15 |
| Provision of infertility services | 8 | 11 |
| Public-Private Partnership | 7 | 8 |
| Staff qualification | 4 | 6 |
| Utilisation of services | 2 | 3 |
| **FACILITATORS TO FERTILITY CARE** | **7** | **10** |
| **FACTORS INFLUENCING FERTILITY CARE** | **43** | **191** |
| Availability of services | 12 | 15 |
| Confidentiality | 7 | 9 |
| Costly treatments | 6 | 6 |
| Drugs, equipment and supplies | 13 | 26 |
| Duration- results of treatment | 2 | 2 |
| Follow-up | 1 | 1 |
| Geographic distance | 5 | 7 |
| Human resources | 4 | 5 |
| Infertility awareness | 4 | 7 |
| Infrastructure | 1 | 1 |
| Lack of standards and guidelines | 7 | 8 |
| Limited public sector | 1 | 1 |
| Men participation in investigation and treatment | 17 | 36 |
| Providers workload | 2 | 3 |
| Repro travel | 3 | 5 |
| Systematic data collection | 9 | 16 |
| Training | 9 | 13 |
| Unaffordable private care | 8 | 10 |
| Unavailable ART | 8 | 19 |
| Cost | 6 | 9 |
| Financial support to individuals | 1 | 1 |
| In private | 1 | 2 |
| Insurance scheme | 1 | 2 |
| Equipment | 1 | 1 |
| Human resources | 1 | 2 |
| **FERTILITY AWARENESS** | **37** | **145** |
| Advocacy | 13 | 16 |
| FertiStat tool | 7 | 9 |
| Importance of sensitisation | 16 | 25 |
| Not implemented | 16 | 26 |
| **FERTILITY POLICY DIALOGUE WORKSHOP** | **20** | **34** |
| Missed attendance | 15 | 18 |
| Missed objectives | 3 | 8 |
| **GOVERNMENT INTERACTION WITH ORGANISATIONS** | **14** | **69** |
| Coordination-collaboration among stakeholders | 7 | 16 |
| Creation of advocacy and awareness | 3 | 4 |
| First Lady office | 5 | 11 |
| Merck Foundation - MTM project | 1 | 1 |
| International partners | 8 | 12 |
| NGOs | 2 | 4 |
| Policymaking | 1 | 4 |
| Private care | 2 | 4 |
| UN agencies | 4 | 14 |
| **HEALTH INFORMATION SYSTEM** | **36** | **181** |
| Capturing infertility data | 35 | 127 |
| Data captured | 6 | 7 |
| Data not captured | 26 | 45 |
| Infertility reported as Other or Infection | 9 | 13 |
| Reporting infertility data | 16 | 22 |
| Data reported | 1 | 1 |
| Data not reported | 9 | 10 |
| **INFERTILITY ALLIANCES** | **4** | **6** |
| National fertility society | 3 | 5 |
| Patients' associations | 1 | 1 |
| Infertile men and women | 2 | 3 |
| Kanyalengs | 3 | 7 |
| **INTEGRATION OF INFERTILITY WITHIN RH SERVICES** | **14** | **22** |
| Family Planning clinic | 4 | 6 |
| Gyn clinic | 4 | 5 |
| Maternal Health clinic | 2 | 3 |
| Standalone | 3 | 3 |
| **LESSONS LEARNED FROM OTHER COUNTRIES** | **3** | **6** |
| **lNFLUENCE OF POLICY CONTEXT** | **45** | **357** |
| Involvement of former government | 10 | 25 |
| New health policy | 45 | 330 |
| Benefits of inclusion | 27 | 43 |
| Challenges of implementation | 42 | 210 |
| Compelling health priorities | 4 | 5 |
| Cost - Funds | 17 | 28 |
| Insurance scheme | 1 | 1 |
| Drugs | 10 | 14 |
| Education-Awareness | 5 | 5 |
| Equipment and Supplies (including lab) | 26 | 35 |
| Fertility training for staff | 24 | 36 |
| Human resources (availability, retention, deployment) | 23 | 46 |
| Infrastructures | 4 | 4 |
| Integration with other services | 1 | 1 |
| Leadership | 1 | 4 |
| Set up ART | 2 | 3 |
| Sustainability | 2 | 11 |
| Treatment | 1 | 1 |
| Other available policies | 1 | 2 |
| **MECHANISMS OF FUNDING FERTILITY CARE** | **9** | **38** |
| Govt | 6 | 12 |
| International partners | 7 | 14 |
| Private | 2 | 7 |
| **PERCEIVED CAUSES OF INFERTILITY** | **38** | **204** |
| Artificial infertility | 9 | 14 |
| Family planning-related | 7 | 13 |
| Gyne-related issues | 10 | 12 |
| Infertility-related myths | 2 | 4 |
| Jujus - evil forces | 4 | 5 |
| Male factor | 9 | 14 |
| Maternal age | 2 | 4 |
| Other causes | 3 | 3 |
| STI-FGM | 30 | 49 |
| Traditional concoctions | 5 | 7 |
| **PERCEIVED IMPORTANCE OF INFERTILITY** | **28** | **99** |
| For health providers (in their communities) | 9 | 12 |
| Big problem | 23 | 40 |
| Lesser problem | 13 | 27 |
| For policy implementers | 1 | 1 |
| For policymakers | 16 | 49 |
| Important | 5 | 5 |
| Not so important | 13 | 19 |
| For women and men | 16 | 24 |
| Other competing factors | 3 | 10 |
| **REFERRALS** | **39** | **148** |
| To private | 18 | 22 |
| To public | 29 | 58 |
| To traditional medicine (referred by health provider) | 17 | 22 |
| To traditional medicine (self-referral) | 13 | 18 |
| **SOCIAL ISSUES OF INFERTILITY** | **33** | **112** |
| Cultural issues | 16 | 26 |
| Community - society | 11 | 17 |
| Family | 5 | 13 |
| Religion | 3 | 3 |
| Spousal issues | 17 | 20 |
| Female problem, stigma and blame | 26 | 44 |
| Women empowerment | 4 | 7 |
| Male dominance-acknowledgment of infertility | 19 | 38 |
| **SUPPORT PROVIDED BY THE HEALTH FACILITY** | **41** | **212** |
| Health services provided | 27 | 57 |
| Infertility investigations | 21 | 47 |
| Infertility services | 14 | 32 |
| Unhealthy practices | 1 | 1 |
| Infertility treatment | 19 | 46 |
| **SUPPORT RECEIVED FROM GOVERNMENT** | **12** | **26** |
| Other support | 7 | 15 |
| **TOP PRIORITES OF RH** | **10** | **33** |
| Men involvement in RH | 1 | 2 |
| Position of infertility among RH priorities | 7 | 10 |
| High priority | 3 | 4 |
| Low priority | 5 | 6 |
| **TRAINING OF HEALTH PROVIDERS** | **19** | **47** |
| Deployment of trained staff | 1 | 2 |
| Fertility management training | 10 | 18 |
| Not received | 18 | 21 |
| Received (notions of infertility) | 16 | 32 |
| Formal training (University or nursing school) | 10 | 21 |
| On-job training | 9 | 11 |
| Other training - Capacity building | 5 | 7 |
| **USE OF TRADITIONAL MEDICINE FOR INFERTILITY** | **11** | **24** |
| Other health issues | 4 | 8 |
